# Role of Dual-Specificity Phosphatase 1 in Glucocorticoid-Driven Anti-inflammatory Responses

**DOI:** 10.3389/fimmu.2019.01446

**Published:** 2019-06-26

**Authors:** Jessica Hoppstädter, Alaina J. Ammit

**Affiliations:** ^1^Department of Pharmacy, Pharmaceutical Biology, Saarland University, Saarbrücken, Germany; ^2^Faculty of Science, School of Life Sciences, University of Technology Sydney, Sydney, NSW, Australia; ^3^Woolcock Emphysema Centre, Woolcock Institute of Medical Research, University of Sydney, Sydney, NSW, Australia

**Keywords:** sepsis, infection, arthritis, bone disease, asthma, COPD, atherosclerosis

## Abstract

Glucocorticoids (GCs) potently inhibit pro-inflammatory responses and are widely used for the treatment of inflammatory diseases, such as allergies, autoimmune disorders, and asthma. Dual-specificity phosphatase 1 (DUSP1), also known as mitogen-activated protein kinase (MAPK) phosphatase-1 (MKP-1), exerts its effects by dephosphorylation of MAPKs, i.e., extracellular-signal-regulated kinase (ERK), p38, and c-Jun N-terminal kinase (JNK). Endogenous DUSP1 expression is tightly regulated at multiple levels, involving both transcriptional and post-transcriptional mechanisms. DUSP1 has emerged as a central mediator in the resolution of inflammation, and upregulation of DUSP1 by GCs has been suggested to be a key mechanism of GC actions. In this review, we discuss the impact of DUSP1 on the efficacy of GC-mediated suppression of inflammation and address the underlying mechanisms.

## Introduction

Glucocorticoids (GCs) are steroid hormones with immunosuppressive activity that are used to treat a wide variety of inflammatory conditions, including rheumatoid arthritis, pulmonary diseases, and acute inflammation caused by microbial infection.

Anti-inflammatory properties of GCs are partially dependent on their ability to suppress mitogen-activated protein kinases (MAPKs) (1, 2). MAPKs are a family of protein kinases that respond to a wide variety of extracellular stimuli. They are activated by phosphorylation of tyrosine and threonine residues within their active domains and are inactivated by dephosphorylation of either residue (2–4). MAPK cascades are evolutionary conserved and control a large number of cellular processes, including proliferation, differentiation, apoptosis, motility, and stress responses. The three major signaling cascades either involve extracellular signal-regulated kinase 1/2 (ERK1/2), c-Jun N-terminal kinase (JNK), or p38 MAPK (2–4). Dysregulation of MAPK activity has been suggested to contribute to the onset of many pathologies, including neurodegenerative diseases, diabetes, cancer, and inflammation (4–7).

**Figure 1:**
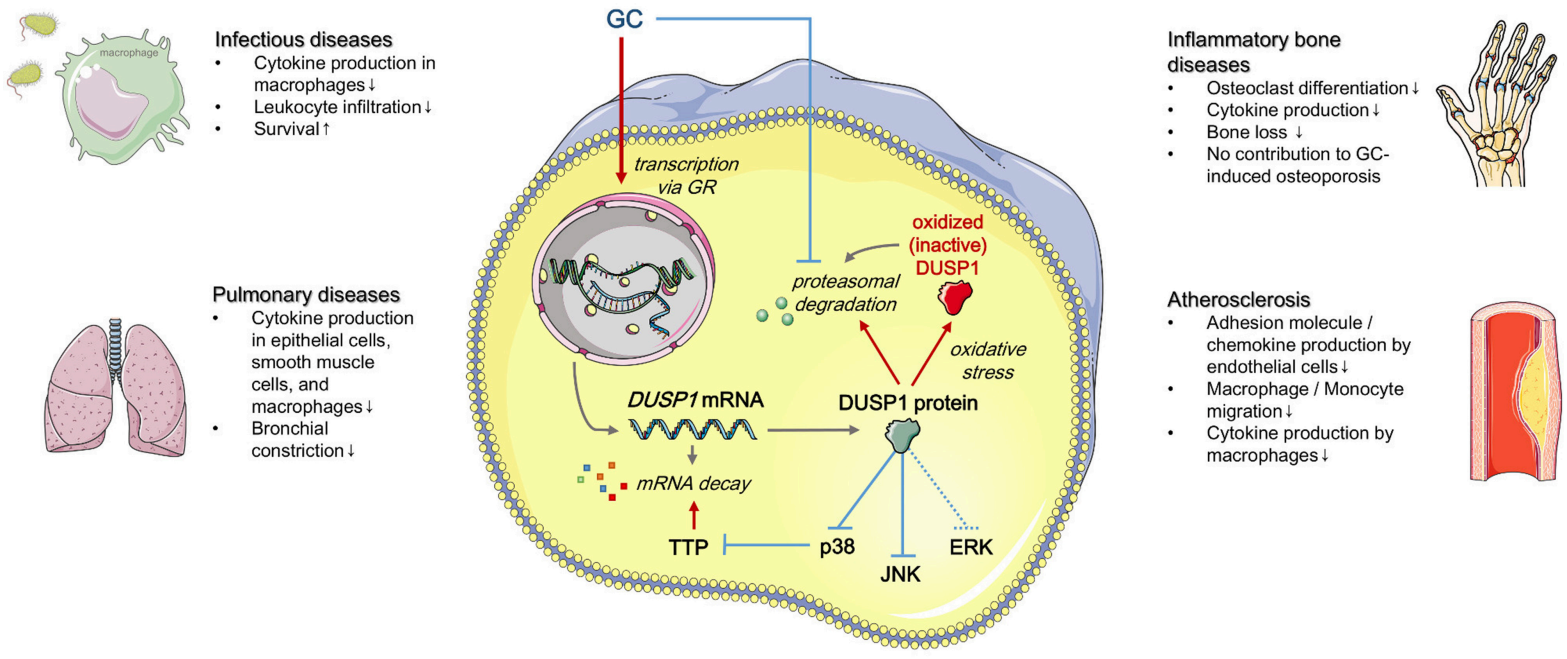
Regulation and effects of GC-induced DUSP1 expression (simplified). Red: positive regulation, blue: negative regulation. See text for details. Templates from Servier Medical Art (http://www.servier.com) were used to generate the figure.

MAPKs can be dephosphorylated by tyrosine-specific phosphatases, serine-threonine phosphatases, or dual-specificity (Thr/Tyr) phosphatases (DUSPs) (8, 9). GC treatment primarily attenuates MAPK signaling via DUSP1, also known as mitogen-activated protein kinase phosphatase-1 (MKP-1) (10–12). In this review, we discuss the influence of DUSP1 on GC-mediated effects (Figure 1).

## Dual-Specificity Phosphatase 1 (DUSP1)

Although DUSP1 was initially identified as an ERK-specific phosphatase, p38 MAPK and JNK are its preferred substrates in several cell types, including myeloid cells (13–16). Thus, DUSP1 activity limits p38 and JNK-dependent pro-inflammatory gene transcription (17–20).

However, DUSP1 is also involved in the regulation of anti-inflammatory genes. Over-production of IL-10 in *Dusp1*^−/−^ mice was observed in peritonitis models after lipopolysaccharide (LPS) challenge or infection with *Escherichia coli* or *Staphylococcus aureus*, and LPS-treated *Dusp1*^−/−^ macrophages. This can be explained by the interaction of DUSP1 with the RNA-binding protein tristetraprolin (TTP, gene name *Zfp36*): increased p38-mediated phosphorylation of TTP results in its inactivation, followed by accumulation of the inactive, but stable, form of TTP and enhanced stability of TTP target mRNAs. These target mRNAs comprise pro-inflammatory chemokines and cytokines, e.g., *Tnf*, *Cxcl1*, and *Cxcl2*, but also the anti-inflammatory *Il10*. Approximately 50% of the genes dysregulated in *Dusp1*^−/−^ macrophages are affected by TTP inactivation (21).

The promoter region of the *Dusp1* gene contains binding sites for several transcription factors, including activator protein 1 (AP-1), nuclear factor-κB (NF-κB), cAMP response element-binding protein (CREB), and the glucocorticoid receptor (GR) (22–25). Hence, DUSP1 can be induced under various conditions, ranging from inflammatory activation to altered cellular metabolism and GC excess during stress responses. GCs may further enhance DUSP1 expression by inhibiting its proteasomal degradation (12).

DUSP1 has been shown in a few studies to be regulated via the stability of its mRNA. Several mRNA binding proteins can influence *Dusp1* mRNA stability. TTP-mediated *Dusp1* mRNA decay has been suggested to be a feedback mechanism in inflammatory responses by which TTP limits its own activity: reduced DUSP1 expression enhances p38 MAPK phosphorylation, thereby promoting TTP inactivation (26). Besides, several miRNAs, such as miR-101, have been shown to modulate DUSP1 expression (27). Posttranslational DUSP1 modifications include phosphorylation, acetylation, and oxidation. ERK-mediated phosphorylation of DUSP1 can either lead to increased or decreased DUSP1 protein stability, depending on the phosphorylation site (28, 29). Acetylation of Lys57 results in increased phosphatase activity and more effective suppression of the MAPK signaling cascades (30, 31). In contrast, oxidation of Cys258 within the active site inactivates DUSP1 and leads to its rapid degradation by the proteasome. In this manner, DUSP1 oxidation prolongs MAPK activation, ultimately resulting in enhanced inflammatory responses (32–34). S-glutathionylation of Cys258 has similar effects, indicating that DUSP1 activity is redox-sensitive (35).

## Role of DUSP1 in Inflammatory Diseases and Its Influence on GC Treatment Efficacy

### Infectious Diseases and Sepsis

In the context of infectious diseases and sepsis, research on the role of DUSP1 focused mainly on macrophage responses. Macrophages are a subtype of innate immune cells with high plasticity that play a crucial role in acute inflammation. They recognize pathogen- or danger-associated molecular patterns via pattern recognition receptors, such as toll-like receptors (TLRs). Stimulation of macrophages initially leads to excessive inflammation, followed by a phenotypic switch toward an anti-inflammatory and wound-healing phenotype that promotes the resolution of inflammation (36, 37).

Early studies on the role of DUSP1 in the response of macrophages to bacterial LPS suggested that DUSP1 is required to balance inflammatory responses in sepsis and infectious diseases. The ectopic expression of DUSP1 in LPS-stimulated macrophages accelerated JNK and p38 inactivation and substantially inhibited the production of TNF-α and IL-6 (38). Moreover, increased cytokine production and elevated expression of the differentiation markers CD86 and CD40 were observed in macrophages from *Dusp1*^−/^^−^ mice when activated by TLR ligands. *Dusp1*^−/−^ macrophages also showed enhanced constitutive and TLR-induced activation of p38 MAPK (39). Moreover, LPS-induced IFN-β production was increased in *Dusp1*^−/−^ macrophages, both due to elevated JNK-mediated activation of cJun and *Ifnb* mRNA stabilization by TTP inactivation (20). DUSP1 induction has also been shown to be involved in endogenous feedback loops initiated by either adenosine or prostaglandin E2 signaling that skew macrophages toward an anti-inflammatory phenotype (40, 41).

Several studies confirmed the relevance of these *in vitro* findings for the *in vivo* situation. In LPS-treated mice, DUSP1 is upregulated in various tissues and cell types and limits p38 MAPK activation. In accordance, depletion of DUSP1 led to the excessive release of inflammatory cytokines, such as TNF-α, IL-6, CCL3, and CCL4, and increased LPS-induced mortality (14, 15, 39, 42). Likewise, *Dusp1*^−/−^ mice showed amplified inflammatory responses and lethality after infection with either *S. aureus* (43) or *E. coli* (44).

The phenotype of *Dusp1*^−/−^ mice in two sophisticated models of sepsis, i.e., caecal ligation and puncture (CLP) and colon ascendens stent peritonitis (CASP), strongly resembled those observed after LPS shock, with highly increased levels of IL-6, CCL3, and CCL4 and excess lethality (45).

Glucocorticoids induce DUSP1 in mouse macrophages, and DUSP1 is required for the inhibition of JNK and p38 MAPK by dexamethasone in these cells (10, 38). Consequently, the GC-mediated shift toward an anti-inflammatory macrophage phenotype was attenuated in cells from *Dusp1*^−/−^ mice (10, 46). In a cutaneous air pouch model, the zymosan-induced production of pro-inflammatory mediators and the infiltration of leukocytes into a pre-formed dorsal cavity were inhibited by oral dexamethasone administration in wild-type, but not in *Dusp1*^−/−^, mice, suggesting that DUSP1 is indeed required to unfold the full anti-inflammatory potential of GCs (10). In another study, the reduction of TNF-α-induced mortality caused by pretreatment with dexamethasone was dependent on the presence of DUSP1: whereas wildtype mice were entirely protected by dexamethasone administration, *Dusp1*^−/−^ animals did not benefit from the GC treatment (1).

In conclusion, both *in vitro* and *in vivo* evidence suggests that DUSP1 critically contributes to the resolution of acute inflammatory responses and mediates protective GC effects in this context.

### Inflammatory Bone Disorders

The bone mass is subject to constant remodeling orchestrated by osteoblasts and osteoclasts. In inflammatory bone disorders, e.g., autoimmune-driven rheumatoid arthritis or pathogen-induced periodontitis, the balance of osteoblast and osteoclast activity is compromised, resulting in bone loss (47).

**Figure 2:**
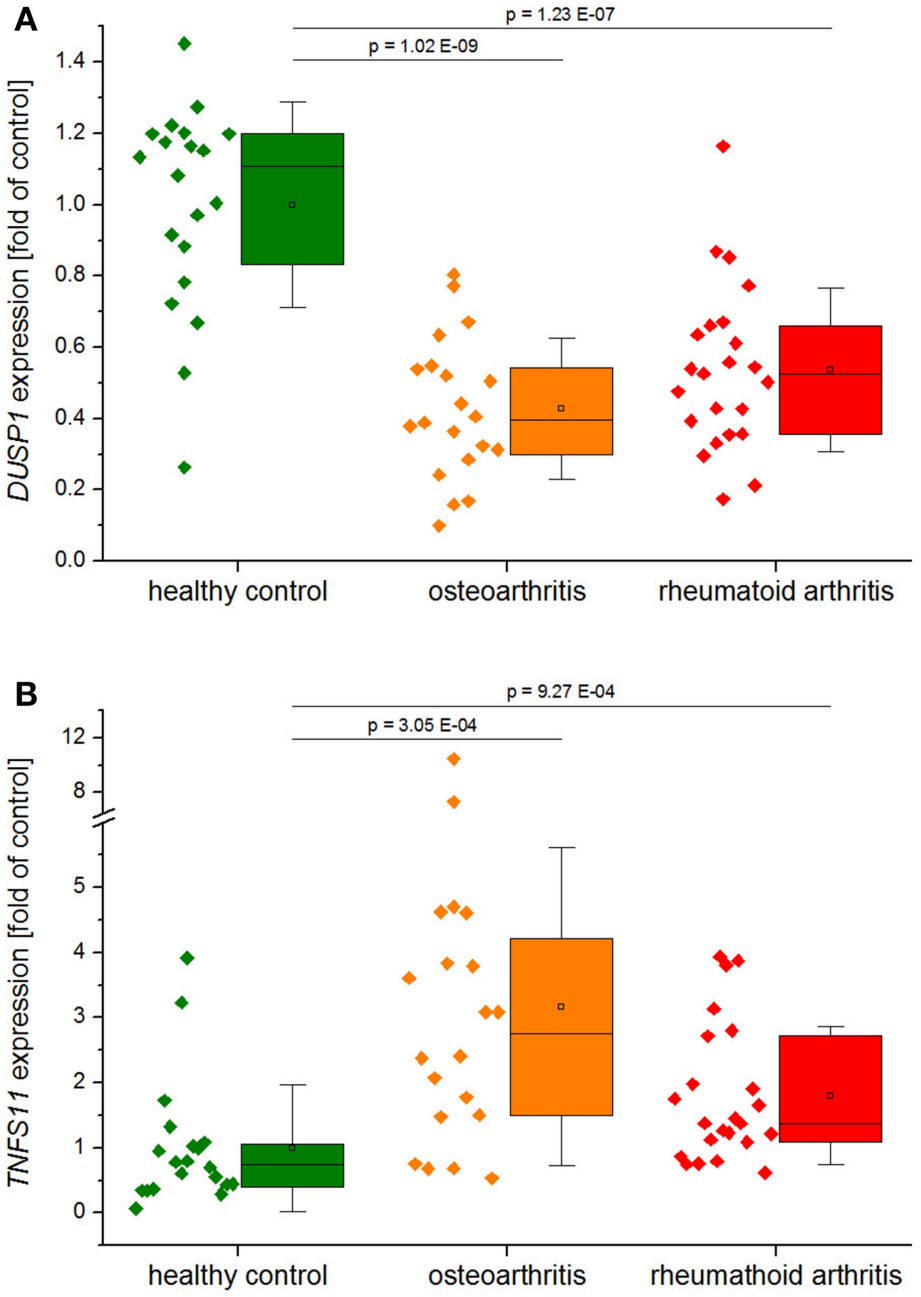
*DUSP1* **(A)** and *TNFSF11* (RANKL, **B**) expression in synovial tissues from healthy controls, patients with rheumatoid arthritis, or osteoarthritis. Data obtained from GEO Datasets GDS5401 (Berlin dataset) and GDS5403 (Jena dataset) were normalized against their respective healthy control values before compilation. Data are shown as individual values per sample, and boxplots show the 25–75th percentiles (box), mean (square), median (line), and standard deviation (whiskers). *P*-values were generated by one-way ANOVA and Bonferroni's *post-hoc* test (**A**, normal distribution) or Mann–Whitney *U*-test (**B**, not normally distributed).

*DUSP1* was strongly downregulated in synovial biopsies from patients with rheumatoid arthritis and osteoarthritis (GEO datasets GDS5401 and GDS5403; Figure 2A), suggesting that DUSP1 deficiency may contribute to disease progression.

DUSP1 indeed effectively reduced osteolysis in studies utilizing mouse models of LPS-induced inflammatory bone loss and collagen-induced arthritis (CIA) (48, 49). *Dusp1*^−/−^ mice showed excessive bone loss, more inflammatory infiltrates, and an increase in osteoclastogenesis at the site of LPS-injection in a model of experimental periodontitis (49). In line with these findings, adenovirus-mediated overexpression of DUSP1 was shown to protect against bone loss in a similar experimental model of periodontal disease (50). Furthermore, *Dusp1*^−/−^ mice exhibited higher penetrance, earlier onset, and increased severity of experimental arthritis, accompanied by higher numbers of osteoclasts in inflamed joints and more extensive loss of bone mass. Complementary *in vitro* experiments showed that DUSP1 acts as a negative regulator of osteoclast formation and activation via suppression of p38 MAPK (48, 51). A recently published study showed that the presence of calcium crystals, which are critical factors in the pathogenesis of osteoarthritis, stimulate receptor activator of NF-κB ligand (RANKL) secretion by osteoblasts via DUSP1 downregulation, thereby promoting osteoclastogenesis (52). RANKL induction was also observed in synovial biopsies from arthritis patients in the GEO datasets mentioned above (Figure 2B). Moreover, overexpression of DUSP1 in fibroblast-like synoviocytes from osteoarthritis patients inhibited the expression of osteoarthritis-associated mediators (53).

However, DUSP1 depletion did not affect age-related spontaneously occurring osteoarthritis, since knockout mice showed a similar disease progression compared to controls at 21 months of age (54). Thus, the modulatory function of DUSP1 in the context of bone homeostasis seems to be most evident in the presence of a potent inflammatory trigger.

Due to their high anti-inflammatory capacity and their ability to decrease radiologic disease progression, GCs are frequently used for the treatment of rheumatoid arthritis. Paradoxically, one common side effect of GC use, primarily when used at high dosages or over prolonged periods, is a loss of bone mass, also known as GC-induced osteoporosis. This adverse effect is associated with increased osteoclastogenesis and depletion of osteoblasts (55, 56).

DUSP1 has been suggested to contribute to GC-induced bone loss since GC-inducible attenuation of osteoblast proliferation involves inhibition of the MAPK/ERK signaling pathway and can be reversed by the protein tyrosine phosphatase (PTP) inhibitor vanadate *in vitro* and *in vivo* (57–59). The assumption that the PTP in question might be DUSP1 was, however, not supported by studies with *Dusp1*^−/−^ mice, which demonstrated that GC-induced bone loss was not prevented upon DUSP1 depletion: after treatment with the GC methylprednisolone for 28 days, both wildtype and *Dusp1*^−/−^ mice showed a similar reduction of osteoid surfaces, volumes, and osteoblast numbers (60).

In summary, loss of DUSP1 favors bone loss, especially under highly inflammatory conditions. Further studies are required to clarify whether DUSP1 contributes to the beneficial or adverse effects of GCs in the therapy of bone-related diseases.

### Pulmonary Diseases

GCs are first line anti-inflammatory medicines in chronic respiratory diseases, including asthma and chronic obstructive pulmonary disease (COPD), and are commonly used therapeutically as inhaled corticosteroids (ICS). ICS effectively control inflammation in asthma but are less effective in COPD. This is thought to be due to corticosteroid insensitivity, where the molecular pathways responsible for the effect of GCs have been modified by oxidative stress or infections (61). Moreover, a subset of asthmatics (~10%) are refractory to ICS and classified as having severe asthma. DUSP1 has been shown to contribute to the effects of GCs in several *in vitro, ex vivo*, and *in vivo* studies with relevance to respiratory disease (61–63) and in some key studies, an impact on DUSP1 function has been shown to be responsible for corticosteroid insensitivity/resistance. For instance, an *ex vivo* study examined the repressive effect of GCs on stimulated production of inflammatory cytokines by alveolar macrophages from patients with severe asthma to those with non-severe asthma. GCs were less effective in macrophages from severe asthma patients, and this GC insensitivity was linked with increased p38 MAPK activation and impaired inducibility of DUSP1 (64).

The first to demonstrate that GCs upregulated DUSP1 in primary airway smooth muscle cells were Issa et al. (65). This was confirmed in a publication by the Ammit group that showed that GC-induced DUSP1 controlled cytokine mRNA stability in a p38 MAPK-mediated manner (66). Notably, knockdown of DUSP1 with siRNA showed that GC-induced DUSP1 was a significant contributor to anti-inflammatory effects at the post-transcriptional level.

Several *ex vivo* and *in vivo* studies utilizing *Dusp1*^−/−^ mice highlighted the contribution of DUSP1 to GC effects in respiratory disease. For example, GC-mediated repression of the contractile response in bronchial rings from mice was abrogated by *Dusp1* depletion (67). Interestingly, the anti-inflammatory impact of DUSP1 was lost in ozone-exposed mice in a model that may recapitulate corticosteroid resistance in severe asthma (68). A plausible explanation is that GC-induced DUSP1 in the wild-type mice was oxidized by ozone and rendered non-functional. Oxidization of DUSP1 may prove to be a roadblock to further development of DUSP1 as a therapeutic target in respiratory disease as oxidative stress is a well-appreciated feature of COPD and other conditions where smoking is a risk factor (69, 70).

Finally, there are publications that note that GC-mediated effects in respiratory disease are DUSP1-independent. These include a study that detected gene expression of known GC targets in biopsies from allergen-challenged asthmatic subjects (71). Evidence from studies utilizing *Dusp1*^−/−^ mice in models with relevance to asthma is somewhat equivocal and does not fully support the assertion that DUSP1 is a significant contributor to the effect of GCs *in vivo* (72).

### Atherosclerosis

GCs are not a therapeutic option for the treatment of atherosclerosis, since side effects of long-term GC treatment include hyperglycemia, hypertension, dyslipidemia, and obesity and may, therefore, promote adverse cardiovascular events (73, 74). However, as inflammation plays a significant role in the pathogenesis in atherosclerosis, GCs may exert some anti-atherosclerotic effects. Early studies demonstrated that dexamethasone reduced the severity of atherosclerosis in experimental rabbit models (75–77). Moreover, vein graft thickening was prevented by short-term dexamethasone treatment in hypercholesterolemic mice (78). The development of a drug-eluting bioadhesive gel that allowed to dissociate the systemic adverse and local anti-inflammatory effects of GC treatment. In atherosclerotic mice, inflamed plaques treated with GC-eluting adhesive gels showed reduced macrophage numbers and developed protective fibrous caps covering the plaque core. This was paralleled by lowered plasma cytokine levels and biomarkers of inflammation in the plaque (79).

The onset of atherosclerosis is triggered by proinflammatory mediators, which induce adhesion molecules in endothelial cells (ECs) by activating MAPKs, particularly p38 MAPK. Dexamethasone-induced DUSP1 upregulation caused inactivation of p38 MAPK in TNF-α-treated ECs and mediated inhibition of E-selectin expression, as shown in murine *Dusp1*^−/−^ ECs and human ECs upon *DUSP1* silencing (80).

The assumption that DUSP1 is atheroprotective via inhibition of EC activation was further supported by studies investigating the influence of shear stress. ECs respond to shear stress via mechanoreceptors that translate mechanical distortions into various molecular signals, including GR translocation (81, 82). Regions of the arterial tree exposed to high shear stress are protected from endothelial activation, inflammation, and atherosclerosis, whereas regions exposed to low or oscillatory shear stress, are susceptible (83, 84). The expression of DUSP1 in cultured ECs was elevated by shear stress, whereas vascular cell adhesion protein (VCAM)-1 levels were reduced; silencing of DUSP1 restored VCAM-1 expression. *In vivo*, DUSP1 was preferentially expressed by ECs in a high-shear, protected region of the mouse aorta and was necessary for the suppression of EC activation (84).

Apart from its effect on the endothelium, DUSP1 also determines the monocyte/macrophage phenotype in atherosclerosis (35, 85, 86).

Metabolic stress was shown to induce the S-glutathionylation, inactivation, and subsequent degradation of DUSP1 in monocytes. As a result, increased p38 MAPK and ERK activity primed monocytes for chemokine-induced recruitment, thereby promoting monocyte adhesion and migration. *In vivo*, transplantation of DUSP1-deficient bone marrow into atherosclerosis-prone mice exacerbated atherosclerotic lesion formation by sensitizing monocytes to chemoattractants and polarizing macrophages toward an inflammatory phenotype (35, 86). Thus, monocyte and macrophage dysregulation by metabolic stress may drive the progression of atherosclerosis due to DUSP1 inactivation.

Interestingly, the administration of inhaled GCs has been suggested to be atheroprotective in asthma patients, although plasma levels of the drug were presumed to be very low and were not sufficient to provoke cardiovascular GC side effects (87). Whether this observation might be due to elevated DUSP1 expression or activity in ECs or the monocyte/macrophage compartment presently remains elusive.

## DUSP1: a Therapeutic Target?

As underscored by this review, there are several clinical areas where targeting DUSP1 (i.e., increasing its amount and/or activity) would be clinically beneficial. These may also comprise psoriasis or colitis, as a number of studies suggested an involvement of DUSP1 downregulation in the pathogenesis of these diseases (88–91).

Novel ligands to upregulate DUSP1 levels might represent an attractive anti-inflammatory strategy—particularly in atherosclerosis, where GCs cannot be used due to their cardiovascular side effects. Corticosteroid-sparing strategies to reduce the GC dose while achieving effective disease control have always been of clinical importance, and this is also a potential area of focus for DUSP1 upregulators. The failure of p38 MAPK clinical trials, including those recently published in COPD (92) could also bolster the search for DUSP1 modulators. The failure of targeting p38 MAPK is because while pro-inflammatory cytokines are repressed, so are the p38 MAPK-driven anti-inflammatory proteins, including DUSP1 (63, 93, 94).

However, there are challenges to overcome in the drive to develop DUSP as a therapeutic target. First and foremost, it is essential to consider that the overall impact of the MAPK-deactivator DUSP1 within the clinical context will depend on the role played by the MAPK involved. If the rationale is that MAPK needs to be inhibited, then there is a need to upregulate DUSP1 (e.g., in respiratory inflammation). Conversely, in some clinical situations, DUSP1 inhibitors may prove beneficial. For example, in some cancers, DUSP1 is overexpressed and is considered responsible for the failure of JNK-driven apoptotic pathways induced by chemotherapeutics; i.e., adjunct therapeutics with a DUSP1 inhibitor would have merit (95). The challenge in drug discovery would, therefore, be developing targeted therapies that could be delivered to the site of disease without collateral damage. Secondly, DUSP1 is sensitive to oxidative stress, and the phosphatase activity can be reduced. Notably, oxidative stress can be cause or consequence of the disease, and GCs themselves can contribute to the production of oxidative stress (96). Thus, although we may find techniques to increase DUSP1 abundance, it may be non-functional due to oxidation. Reactivation of oxidized DUSP1 function is worthy of further investigation. Thirdly, and perhaps most importantly, we need to get the timing right and ensure that that the temporal kinetics of the impact on DUSP1 on inflammatory pathways are considered. Taken together, the future utility of DUSP1 as a therapeutic strategy depends on it being active (not oxidized) and present at the right place at the right time. Treatment with exogenous DUSP1 upregulators would be akin to the usage of p38 MAPK inhibitors and as they have failed in clinical trials, restoring physiological DUSP1 activity in a manner that fully exploits dynamic regulation exerted by the p38 MAPK/DUSP1/TTP network might even be the better option.

## Author Contributions

JH and AA reviewed the literature and drafted the manuscript.

### Conflict of Interest Statement

The authors declare that the research was conducted in the absence of any commercial or financial relationships that could be construed as a potential conflict of interest.

## Abbreviations

AP-1: activator protein-1
CASP: colon ascendens stent peritonitis
CCL: CC-chemokine ligand
CLP: caecal ligation and puncture
COPD: chronic obstructive pulmonary disease
CREB: cAMP response element-binding protein
CXCL: C–X–C motif ligand
DUSP1: dual-specificity phosphatase
ERK: extracellular-signal-regulated kinase
EC: endothelial cell
GC: glucocorticoid
GR: GC receptor
GRE: GR responsive element
ICAM1: intercellular adhesion molecule 1
ICS: inhaled corticosteroid
IL: interleukin
INF: interferon
IRFs: interferon regulatory factors
JNK: c-Jun N-terminal kinase
LPS: lipopolysaccharide
NF-κB: nuclear factor-κB
MAPK: mitogen-activated protein kinase
MKP-1: mitogen-activated protein kinase phosphatase-1
RANKL: receptor activator of NF-κB ligand
TNF: tumor necrosis factor
TLR: toll-like receptor
TTP: tristetraprolin
VCAM-1: vascular cell adhesion molecule 1.
